# Harvard

**Published:** 1895-11

**Authors:** 


### HARVARD VETERINARY DEPARTMENT.

Professor C. P. Lyman has been granted a year's leave of absence from official duty, with the object of regaining his health, which is much impaired. Professor Osgood has been elected acting dean for 1895–96. Dr. A. J. Sheldon has been appointed instructor in meat inspection for one year from September 1st.

The following reappointments have been made for the sessions of 1895 and 1896: Dr. L. H. Howard, clinical lecturer; W. O. Underwood, A.B., lecturer on warranty and evidence; W. L. LaBaw, D.V.S., demonstrator of anatomy and assistant surgeon; Dr. George B. Foss, house surgeon and lecturer on canine pathology; F. I. Proctor, A.M., M.D., instructor in ophthalmology.

The many friends of Dr. Theobald Smith will be glad to learn that veterinary science is still to participate in his teaching and laboratory work, he having accepted a professorship in Harvard University, and students of the third class in the veterinary department will have the benefit of his great experience and instruction upon the pathology and etiology of the infectious diseases of domestic animals, giving with this course a number of practical exercises.

